# The 2021 Tower Lake Dense Array Experiment for High-Resolution Microseismicity Monitoring During Hydraulic Fracturing

**DOI:** 10.1038/s41597-026-07358-1

**Published:** 2026-05-09

**Authors:** Ramin M. H. Dokht, Honn Kao, Ryan Visser, Chet Goerzen, Stuart Venables

**Affiliations:** 1 Natural Resources Canada, Geological Survey of Canada, Sidney British Columbia, Canada; 2 School of Earth and Ocean Sciences, University of Victoria, British Columbia, Canada; 3 Alberta Geological Survey, Alberta Energy Regulator, Calgary, Alberta Canada; 4 British Columbia Energy Regulator, Victoria British Columbia, Canada

## Abstract

The rapid expansion of hydraulic fracturing (HF) operations in the Western Canada Sedimentary Basin (WCSB) has significantly altered regional seismicity patterns, leading to an increased frequency of injection-induced earthquakes (IIE). While existing regional networks provide robust monitoring for moderate-magnitude events, they often lack the resolution required to characterize small-magnitude seismicity and detailed rupture processes. To address this, a collaborative research project was launched to deploy the Tower Lake Dense Array (TLDA), a temporary high-resolution seismic network in northeast British Columbia. The TLDA (network code 4R) consisted of 36 stations, comprising 33 strong motion and 3 weak motion sensors, deployed in a 6 × 6 km grid with approximately 1 km inter-station spacing. Operating from January to June 2021, the array utilized novel high-resolution sensors, featuring 24-bit digitization, real-time wireless transmission, and GPS time synchronization. Technical validation using teleseismic and local injection-induced events demonstrates that the array provides high-fidelity waveforms across a broad frequency band. Analysis identified a consistent 180^∘^ polarity inversion in the vertical channels of the TLDA that must be corrected. This paper introduces the publicly available TLDA dataset and evaluates its operational performance.

## Background & Summary

The development of unconventional hydrocarbon resources has significantly altered the seismic pattern in North America, in particular the continental interiors of the U.S. and Canada where the historical seismicity was relatively infrequent^1,2^. In western Canada, wide-spread operations of hydraulic fracturing (HF) primarily target the tight siltstone and shale formations with high production potential, including the Montney Play in British Columbia (BC)^3,4^ and the Duvernay Play in Alberta (AB)^5,6^. Detailed investigations of regional seismicity in the Western Canada Sedimentary Basin (WCSB) have shown that the seismogenic response to fluid injection varies significantly across the region^7–9^. Although the vast majority of HF operations were completed without detecting any injection-induced earthquakes (IIE), some have been linked to moderate-size events that generated ground shaking strong enough to raise safety concerns for local communities^10,11^. As a result, most regulatory agencies now require operators to deploy real-time seismic monitoring during injections in IIE-prone areas as part of the safety plan to mitigate the induced seismic risk.

**Fig. 1:**
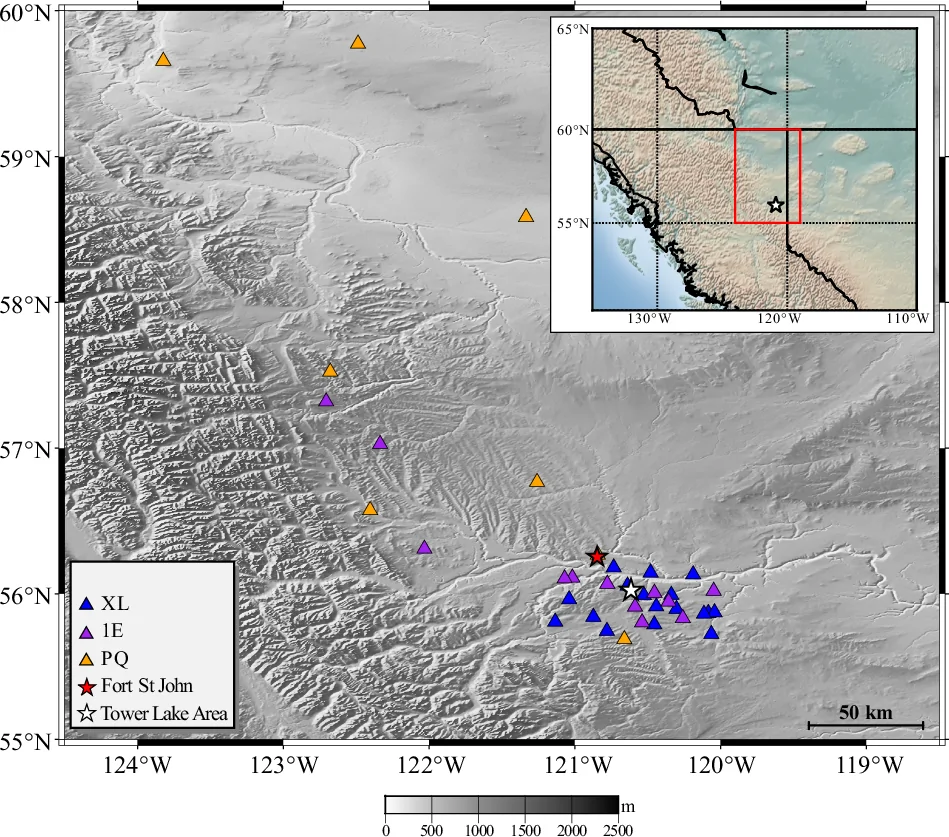
Map of northeast British Columbia showing the locations of permanent seismic stations belonging to 3 different networks (XL stations: blue triangles; 1E stations: magenta triangles; PQ stations: orange triangles). Red and white stars show the locations of Fort St. John and the Tower Lake experiment, respectively. The inset map illustrates the location of the study area in western Canada.

A robust real-time seismograph network with stations located near injection sites is essential to understanding the physical mechanisms linking fluid injection to induced seismicity and to characterizing the temporal and spatial evolution of seismic patterns. Before 1999, the only seismic station in the WCSB was operated by the Canadian National Seismograph Network (CNSN) in Fort St. James, BC (station code FSJ), limiting event detection to magnitudes above M3^12^. In 1999, a second station was established in Fort Nelson, BC, which improved the regional completeness to approximately M2.5. However, accurate hypocentral locations and depth estimates remained limited. In response to the growing frequency of IIEs, Natural Resources Canada (NRCan) launched the Induced Seismicity Research (ISR) Project in 2012, under the Environmental Geoscience Program, and partnered with academic institutions, and provincial and territorial agencies to densify the WCSB’s real-time seismograph network, covering northeast BC, west Alberta, southeast Yukon Territory, and central Northwest Territories^13^. As of August 2025, the network includes 37 stations operated by 3 agencies (Geological Survey of Canada of Natural Resources Canada, BC Energy Regulator, and McGill University), providing real-time waveform data for regional seismic monitoring^14^. The highest station density is in the Fort St. John area, where HF activity and IIE occurrence are concentrated and the magnitude of completeness reaches approximately M1^15^ (Fig. 1).

While the expanded regional network provides robust coverage for the detection and location of IIE, it lacks the resolution necessary to resolve the detailed rupture processes of small-magnitude events − particularly the majority of IIE within the WCSB with M <~2.5. To capture such events and improve the accuracy of source location, dense temporary arrays deployed in close proximity of HF operations are an effective supplement. In addition to improving detection thresholds and event characterization, dense array recordings enable advanced waveform processing techniques such as beamforming and waveform decomposition, which can reveal subsurface structure and fault zone properties^16–18^. The number and configuration of instruments in such deployments can vary significantly depending on scientific objectives, ranging from a few dozen stations^19^ to several hundred or more^20–22^. Most arrays are configured in grids with interstation spacing < 10 km and sampling rates > 200 Hz to ensure high-resolution data capture^23^.

After 6 years of extensive efforts to densify the WCSB regional network, NRCan and the BC Oil and Gas Commission (now the BC Energy Regulator, BCER) agreed to further enhance induced seismic risk mitigation by investigating the physical mechanisms linking the fluid injection and its geomechanical effects to the seismogenesis of IIE. Around the same time, a Calgary-based startup, Symroc Business and Project Management Ltd. (Symroc), developed two prototypes of high-resolution broadband seismic sensors with long-range wireless data transmission capabilities. Seeking real-world testing opportunities, Symroc contacted NRCan, proposing the use of their instruments, especially to detect small-magnitude induced events. In 2020, Symroc received a grant from the Innovation Solutions Canada-Testing Stream (ISC-TS) to manufacture 30 sensor units (including weak and strong motion sensors). The ISR Project at NRCan was appointed by ISC-TS as the technical lead to oversee the testing process. With full support from BCER and the energy industry, a deployment plan was developed for a selected HF site to satisfy the funding requirements. In addition to addressing scientific objectives set by NRCan and BCER, NRCan was also responsible for evaluating the product performance and providing feedback to ISC-TS and Symroc prior to potential commercialization.

Following a vigorous site selection process led by NRCan and BCER, several potential sites were identified for the deployment of Symroc instruments. Taking into account logistical considerations and the operator’s injection schedule, a consensus was reached to deploy all available units as a dense seismic array surrounding a HF site in the Tower Lake area (see Fig. 1). A key factor in site selection was the proximity to existing seismograph stations equipped with broadband sensors from other manufacturers. Since the Tower Lake experiment represents the first scientific application of Symroc instruments, direct comparison of waveform data was essential to evaluate the system’s performance and reliability.

After the completion of the Tower Lake Dense Array (TLDA; network code 4R) experiment, the collected dataset was rigorously reviewed to assess waveform quality, signal consistency, and operational reliability. Particular attention was given to calibration and comparison with nearby permanent stations for both regional and teleseismic events. Following quality control and formatting in accordance with the Federation of Digital Seismograph Networks (FDSN) standards, the full TLDA dataset was archived and made publicly available. This paper introduces the TLDA dataset, outlines the technical specifications of the deployment, and presents key indicators of data quality. We also demonstrate its utility through preliminary seismological analyses relevant to the source study of IIE and highlight ongoing and planned research applications using the dataset.

## Methods

### Instrument Characteristics

**Fig. 2:**
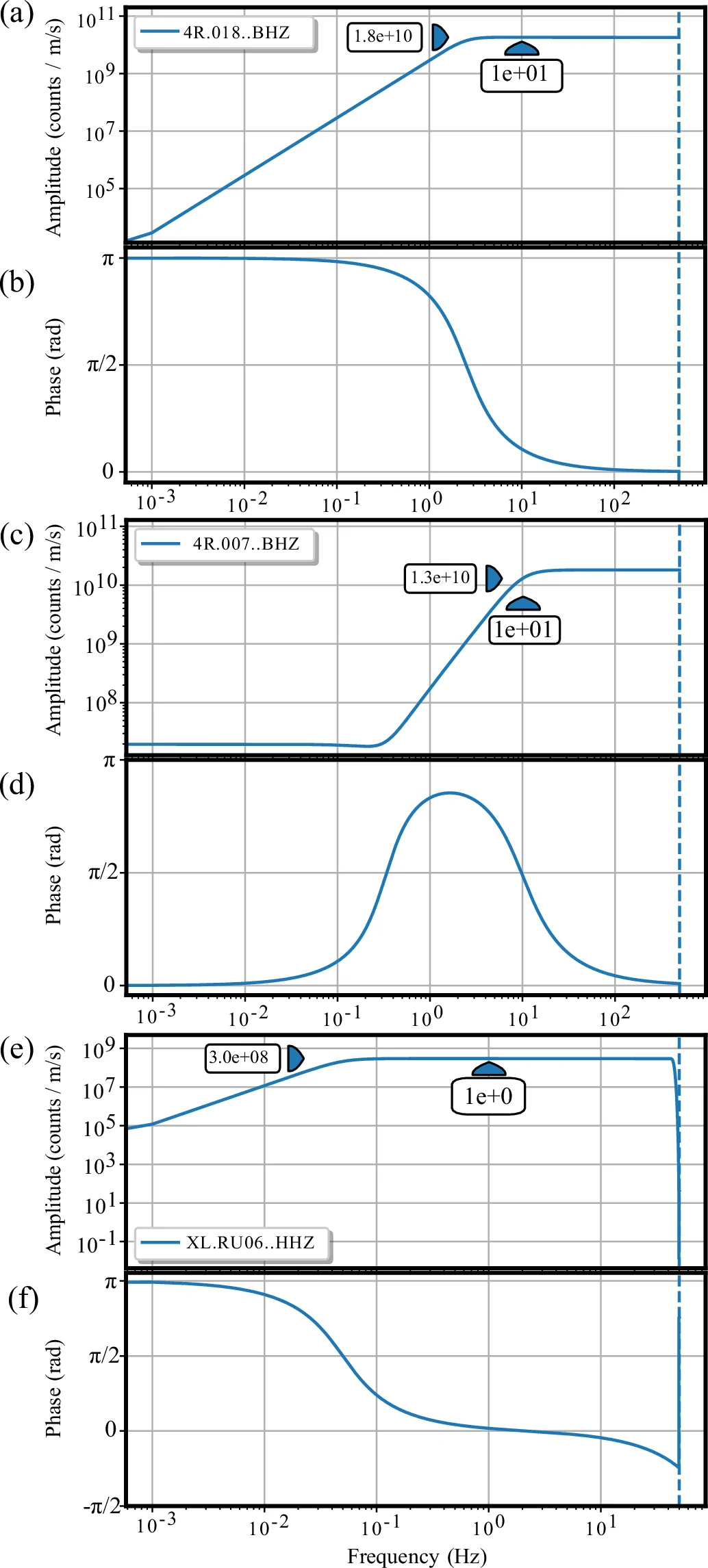
Amplitude and phase responses of a Symroc weak motion sensor (panels a and b, respectively); Amplitude and phase responses of a Symroc strong motion sensor (panels c and d, respectively); Amplitude and phase responses of a Nanometrics Trillium Compact 20S broadband sensor deployed at station XL.RU06 (panels e and f, respectively). The instrument response curves were generated using the plot_response function from the open-source Python library Obspy^29^. Listing S1 of Supporting Material shows the source-code used to download and plot the instrument response curves for 4R.018..BHZ. The instrument response curves of 4R.007..BHZ and XL.RU06..HHZ can be visualized in a similar way.

The instrument response, characterized by transfer functions provided by manufacturers, is crucial to understand how a seismic sensor modifies the ground motion before it is digitally recorded. The instrument response of the weak motion sensor exhibits characteristics typical of a broadband velocity seismometer (see Listing S1 of Supporting Material for instructions on obtaining and visualizing the instrument response curves). The amplitude response is primarily governed by two zeros at the origin and two complex conjugate poles approximately located at $-10.2102\pm 11.9370i\,\frac{{\rm{rad}}}{{\rm{s}}}$ in the Laplace domain, generating a classic flat-to-velocity transfer function^24^. These poles define a corner frequency of approximately 2.5 Hz (corresponding to a period of ~0.4 s; Table S1 of Supplementary Material), below which the instrument sensitivity to ground velocity significantly decreases. Within its operational bandwidth, extending from the given corner frequency up to high frequencies (below its Nyquist frequency of 500 Hz), the sensor demonstrates a relatively flat amplitude response to ground velocity, with a total sensitivity of approximately $1.8\times 1{0}^{10}\,\frac{{\rm{counts}}}{{\rm{m}}/{\rm{s}}}$ over its passband. This design ensures fidelity in recording the amplitudes of seismic signals within its specified frequency range (Fig. 2a).

The phase response of this instrument is similarly defined by its poles-zeros configuration. The two zeros at the origin contribute a constant phase lead of *π* radians across all frequencies. The complex conjugate poles at $-10.2102\pm 11.9370i\,\frac{{\rm{rad}}}{{\rm{s}}}$ introduce a frequency-dependent phase shift, starting at 0 radians at very low frequencies and rolling off and approaching *π* radians phase delay at high frequencies. At very low frequencies (well below the corner frequency of ~2.5 Hz), the combined effect of the poles and zeros results in a phase response approaching a flat *π* radians. As frequencies increase and enter the instrument’s flat-amplitude passband, the phase response transitions from *π* radians to 0 radians. Within this linear phase region, which extends from the corner frequency up to higher frequencies, all frequency components experience a constant phase delay to compensate for the *π*-radian phase lead of double zero in the response. This is crucial for preserving the temporal relations and waveform shapes of incoming seismic signals. At frequencies slightly above the corner frequency, the phase response asymptotically approaches 0 radians. Deviations from this linear phase behavior occur outside the instrument’s optimal flat-response bandwidth, particularly at very low frequencies (see Fig. 2b).

The response of the strong motion sensor is governed by its unique poles-zeros configuration. Unlike the weak motion sensor, this instrument features two complex conjugate zeros at $-1.1404\pm 1.7317i\,\frac{{\rm{rad}}}{{\rm{s}}}$ (at an undamped natural frequency of ~0.3 Hz) and two complex conjugate poles at $-43.9823\pm 44.8709i\,\frac{{\rm{rad}}}{{\rm{s}}}$ (at an undamped natural frequency of ~10 Hz; see Table S1 of Supplementary Material). The presence and location of the zeros, off the origin, alter the low-frequency behavior compared to a typical broadband velocity sensor. This configuration suggests a different bandpass characteristics, optimized for capturing the higher frequency content typical of strong ground motions, while attenuating very long-period signals. The sensor demonstrates a relatively flat amplitude response to ground velocity within its primary operational bandwidth, with a total sensitivity of approximately $1.29\times \,1{0}^{10}\,\frac{{\rm{counts}}}{{\rm{m}}/{\rm{s}}}$ at 10 Hz (Fig. 2c). Overall, the high sample rate of 1000 Hz (in both strong motion and weak motion sensors) further underscores the capability to faithfully record high-frequency seismic energy up to the Nyquist frequency of 500 Hz, which is crucial for engineering seismology applications.

The phase response of the strong motion sensor exhibits a characteristic hump shape, which is a direct consequence of its specific pole and zero locations. At very low frequencies, both the zeros and poles contribute minimally to the phase, resulting in a phase response that approaches 0 radians. As the frequency increases and approaches the zeros’ corner frequency of ~0.3 Hz, the complex conjugate zeros introduce a significant phase lead, causing the total phase response to increase, peaking at a value slightly less than *π* radians. The positive phase shift dominates the response until the influence of the poles becomes more prominent. As the frequencies continue to increase and approach the poles’ corner frequency of ~10 Hz, the phase lag introduced by these complex conjugate poles causes the overall phase response to decrease rapidly. Ultimately, at very high frequencies approaching the Nyquist frequency of 500 Hz, the phase response asymptotically returns to 0 radians (see Fig. 2d). The well-engineered phase response preserves the waveform shapes and maintains the temporal integrity of seismic recordings across a sensor’s intended bandwidth.

A comparative analysis of the Symroc weak and strong motion sensors and the Nanometrics Trillium Compact 20S (a broadband sensor which some of the nearby permanent stations are equipped with) reveals distinct characteristics in their amplitude and phase responses. Regarding amplitude response, the Symroc weak motion sensor exhibit a flat-to-velocity transfer function governed by two zeros at the origin and two complex conjugate poles, yielding a flat amplitude passband above ~2.0 Hz. Conversely, the Symroc strong motion sensor, designed for higher-frequency events, employs complex conjugate zeros off the origin and different pole locations, which significantly alter its low-frequency attenuation and optimize its response for strong motion signals. The Nanometrics broadband sensor is characterized by a more intricate multi-stage response with numerous poles and zeros, including those at very low frequencies. The natural frequency of the dominant poles at approximately 0.05 Hz (i.e., 20 s) demonstrates a much broader amplitude bandwidth that extends to significantly lower frequencies compared to the Symroc sensors. The poles with higher natural frequencies greater than ~55 Hz define the high frequency roll-off of the Nanometrics broadband sensor, and the zeros at higher frequencies (with natural frequencies >~60 Hz) introduce notches, making the instrument significantly less responsive at frequencies close to the Nyquist frequency of 100 Hz. The overall sensitivities of the Symroc sensors are higher than that of the Trillium Compact 20S sensor ($2.98993\times 1{0}^{8}\,\frac{{\rm{counts}}}{{\rm{m/s}}}$ at 1 Hz), which affect both their saturation levels and their ability to resolve very small-amplitude signals (see Fig. 2a,c,e).

Regarding the phase response, both the Nanometrics and Symroc weak motion sensors exhibit a phase lead of *π* radians at very low frequencies below their respective corner frequencies of approximately 0.05 and 2 Hz, due to two zeros at the origin. Within their operational passbands, both sensors are designed to minimize phase distortion, resulting in relatively flat phase responses. In contrast, the distinct poles-zeros configuration of the Symroc strong motion sensor suggests a different phase characteristic, optimized for very high frequencies (see Fig. 2b,d,f).

### Deployment and Data Acquisition

**Fig. 3:**
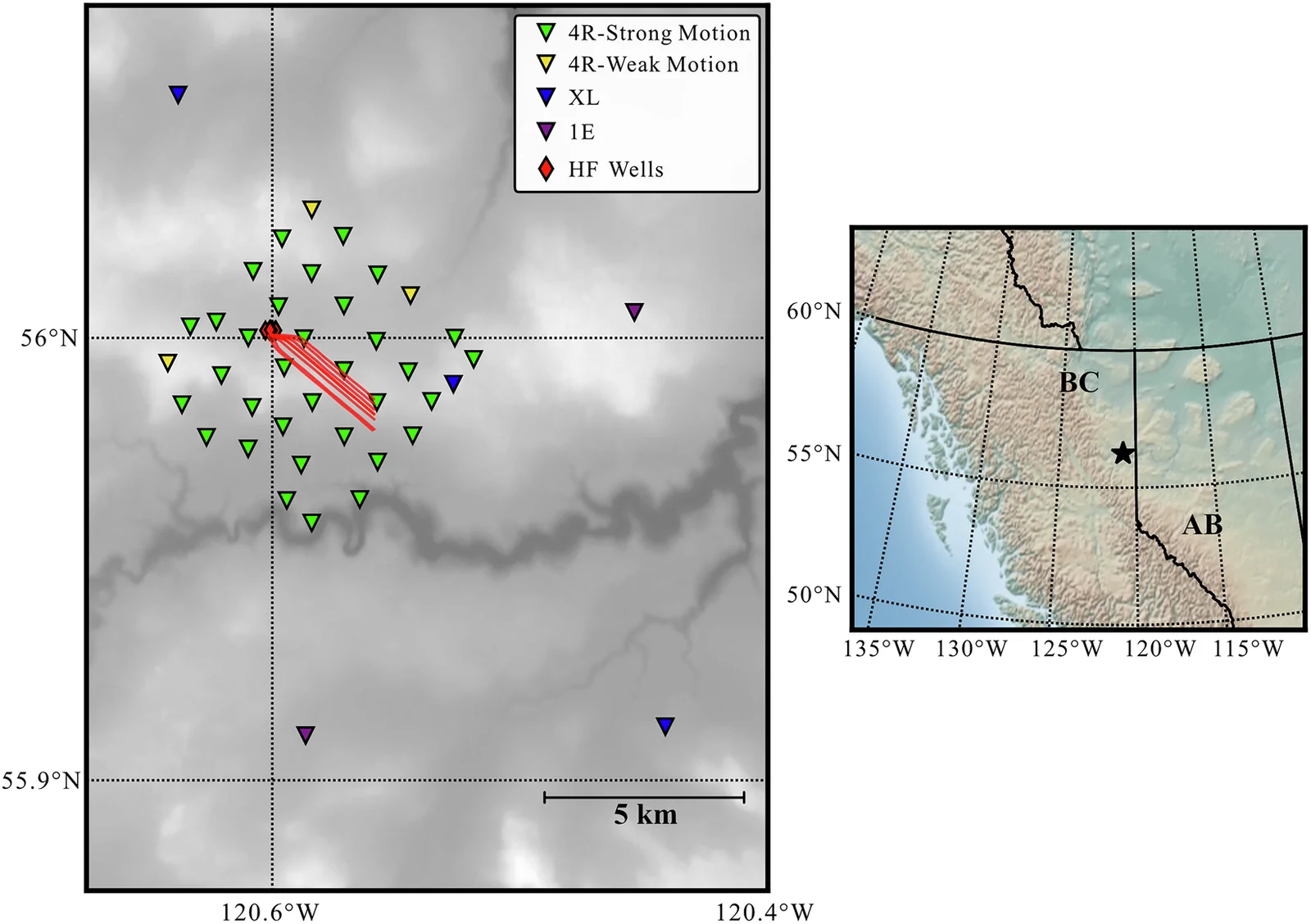
Map of the Tower Lake Dense Array (TLDA) study area showing the locations of 36 Symroc stations (network code 4R) and permanent seismic stations (network codes XL and 1E). Red diamonds indicate the wellhead locations of HF wells active during the experiment, and red lines represent their horizontal sections. The map on the right displays the location of the study area within western Canada. The provinces of Alberta (AB) and British Columbia (BC) are marked on the map. The TLDA station coordinates are given in Table S2.

In late 2020, a dense seismic array was deployed in the Tower Lake area, located in northeast British Columbia, approximately 40 km southeast of Fort St. John. The project installed 36 temporary monitoring stations, laid out on a grid of roughly 6 × 6 km, with approximately 1 km spacing between the stations (Fig. 3). The temporary deployment of the Tower Lake array augmented the existing local networks (1E, PQ and XL^25–27^) and was in operation from 26 January 2021 to 6 June 2021. This project was a collaborative effort between government, industry and local stakeholders, involving Natural Resources Canada, Innovation Solutions Canada, the BC Energy Regulator and Symroc, and its primary goal was to improve understanding of injection-induced seismicity in this area. Thirty-three stations of the TLDA host strong motion sensors and 3 stations host weak motion sensors (see Fig. 3), which are being field-tested through this project. Each station featured GPS timing and standalone solar-battery power, and its compact, weatherproof design allowed quick and easy installation and relocation, making it suitable for short-term induced seismicity monitoring.

Data from the Tower Lake array were collected at high resolution in real time. Each device included an ultra-wideband Analog-to-Digital Converter (ADC) capable of sampling from 0.001 to 1600 Hz with a dynamic range of over 120 dB, potentially effectively capturing both microseismic and low-frequency seismic signals. The Symroc sensor system recorded continuous three-component ground motion with 24-bit digitization. In addition to local storage, it simultaneously transmitted data wirelessly via an integrated communication module over the cellular network. The data were time-synchronized across the array using GPS to an accuracy on the order of one microsecond and archived in a consistent format. In this project, the incoming data feeds were integrated into the routine monitoring system established by NRCan and BCER. All recorded data and detected event catalogs were planned to be made public after the study, following quality control, in order to inform both regulators and the scientific community.

## Data Records

The dataset has been archived at the EarthScope SAGE Data Management Center and is publicly available. To prepare the waveform data and metadata for submission to NSF’s Seismological Facility for the Advancement of Geoscience (SAGE), the dataset must comply with the FDSN standards and use a specifically allocated network code (the Tower Lake array has been assigned the network code 4R^28^). Waveforms are archived in miniSEED format, while metadata, including station inventory, channel responses, and digitizer calibrations, are prepared in StationXML or dataless SEED inventories. Once validated, the miniSEED records are reliably streamed to the SAGE Data Management Center via the miniseed2dmc client over a TCP connection, using state and SYNC files to manage resumable, record-level transfer. This ensures the data’s interoperability, discoverability, and long-term archival integrity.

## Data Overview

**Fig. 4:**
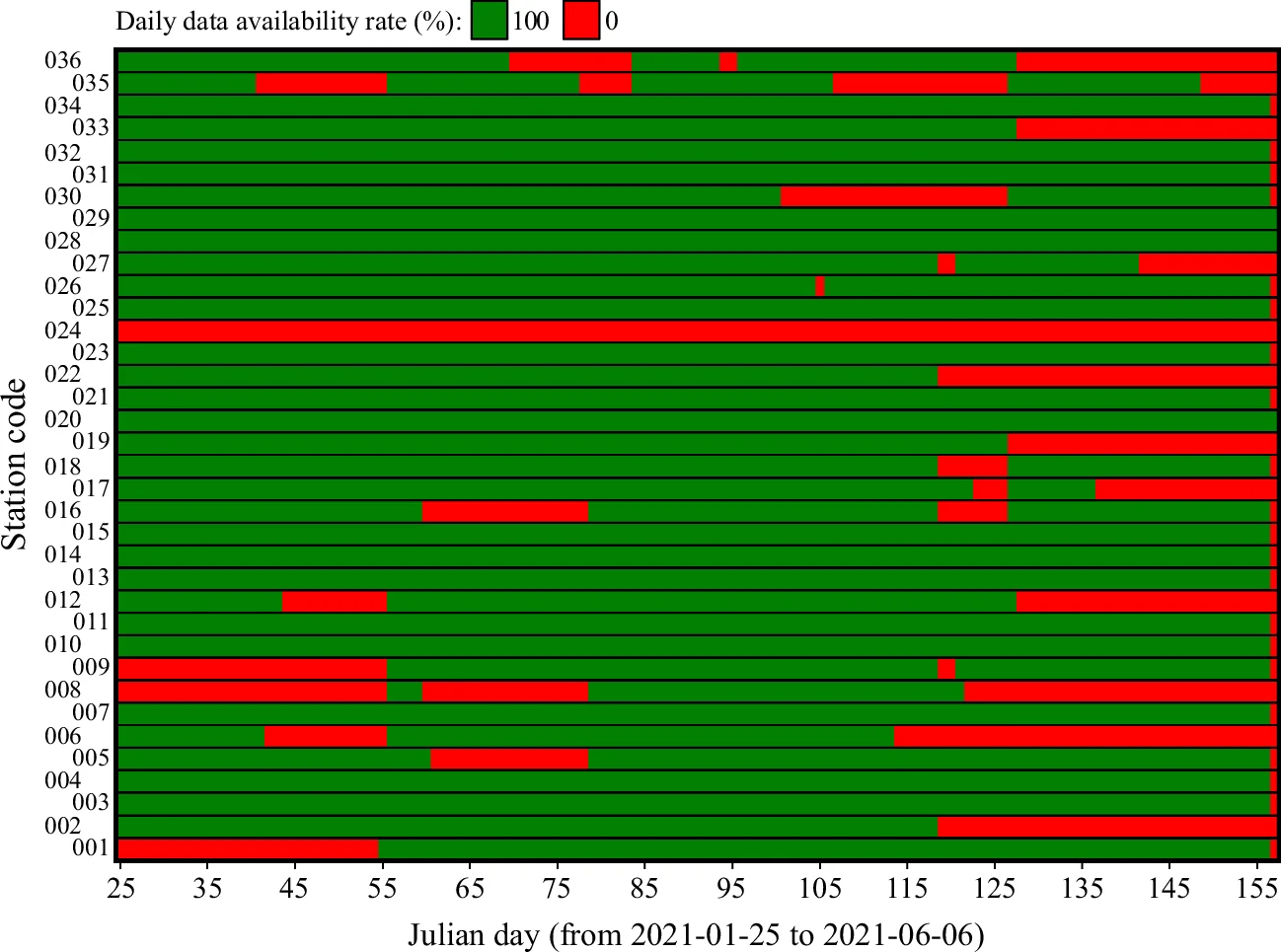
A color-coded matrix showing the daily data availability rates for the TLDA stations. Green and red colors indicate full and missing data for each station over the 133-day operational window.

The data availability assessment of the TL array, comprising 36 stations, reveals variability in recording performance over the 133-day deployment period (Fig. 4). Station 4R.26 remained offline throughout the experiment, yielding no data. Conversely, stations 4R.28 and 4R.29 achieved complete data capture with a data availability rate of 100%. The daily data availability at each station exhibited a binary pattern; days were either fully recorded (100% availability) or entirely missing (0% availability). Overall, approximately 83% of the stations maintained total data availability rates exceeding 70%, while ~61% and ~53% of the stations exceeded the thresholds 80% and 90%, respectively (Table 1).

**Table 1:** Station-level data availability across the 133-day deployment period of the Tower Lake Dense Array.

Total Data Availability Rate			
Station code	Data availability	Station code	Data availability
4R.001	76.69%	4R.019	76.69%
4R.002	70.68%	4R.020	100%
4R.003*	99.25%	4R.021	99.25%
4R.004	99.25%	4R.022	70.68%
4R.005	85.71%	4R.023	99.25%
4R.006	56.39%	4R.024	0%
4R.007	99.25%	4R.025	99.25%
4R.008	35.34%	4R.026	98.50%
4R.009	74.44%	4R.027	86.47%
4R.010	99.25%	4R.028	100%
4R.011	99.25%	4R.029	100%
4R.012	68.42%	4R.030	79.70%
4R.013	99.25%	4R.031	99.25%
4R.014	99.25%	4R.032	99.25%
4R.015	99.25%	4R.033	77.44%
4R.016	78.95%	4R.034	99.25%
4R.017	81.20%	4R.035*	62.41%
4R.018*	93.23%	4R.036	65.41%

Percentage values reflect the proportion of total days during which each station successfully recorded usable waveform data. Stations marked with * denote those equipped with weak motion sensors.

## Technical Validation

### Waveform Accuracy

**Fig. 5:**
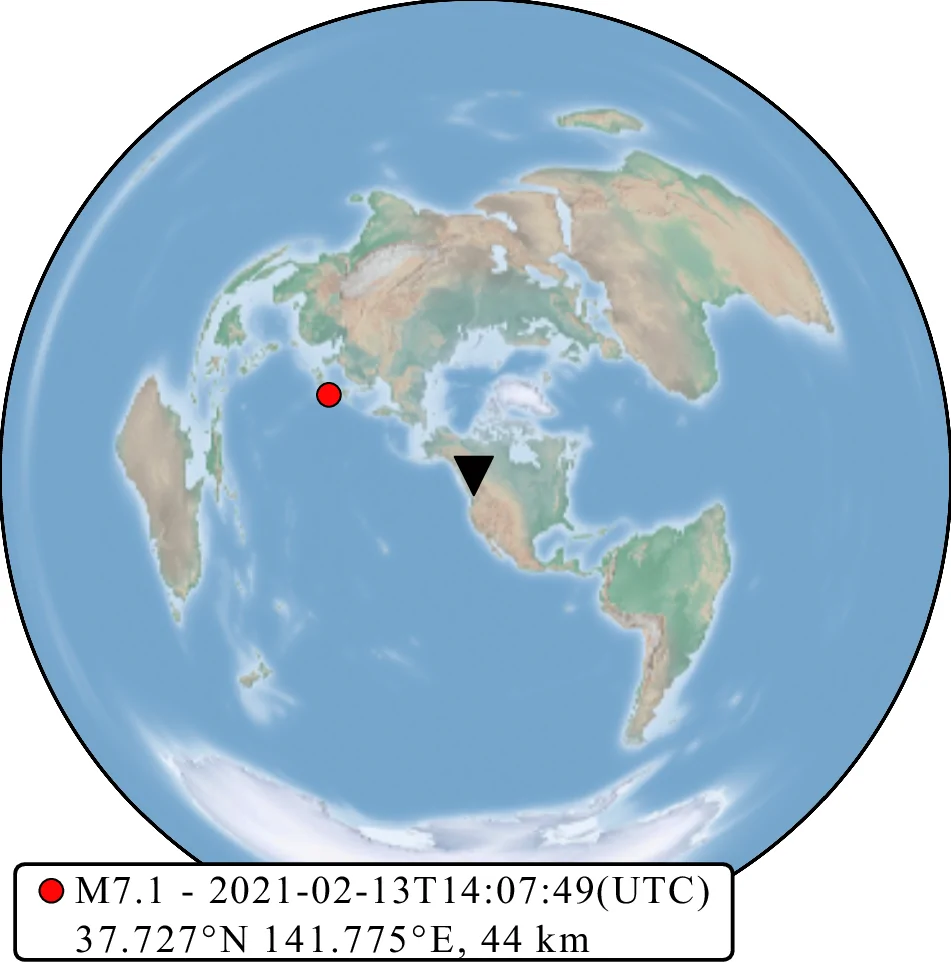
Global map showing epicenter of the *Mw* 7.1 Japan teleseismic event (red circle) and the location of recording stations in Canada (black triangle). Epicentral distances to stations XL.RU06, 4R.018, and 4R.007 are approximately 7048, 7043 and 7046 km, respectively.

A stringent broadband benchmark for evaluating the fidelity of Symroc sensors is provided by a teleseismic test event, as the source is well-constrained. The station XL.RU06, which is equipped with a Trillium Compact 20S and deployed near the TL array, is used as the reference broadband station due to its thoroughly characterized response. We rely on an *Mw* 7.1 teleseismic event that occurred 73 km ENE of Namie, Japan, on February 13, 2021, and focus on the *P*-wave window for a direct sensor-to-sensor comparison of broadband amplitude and phase behavior (Fig. 5).

**Fig. 6:**
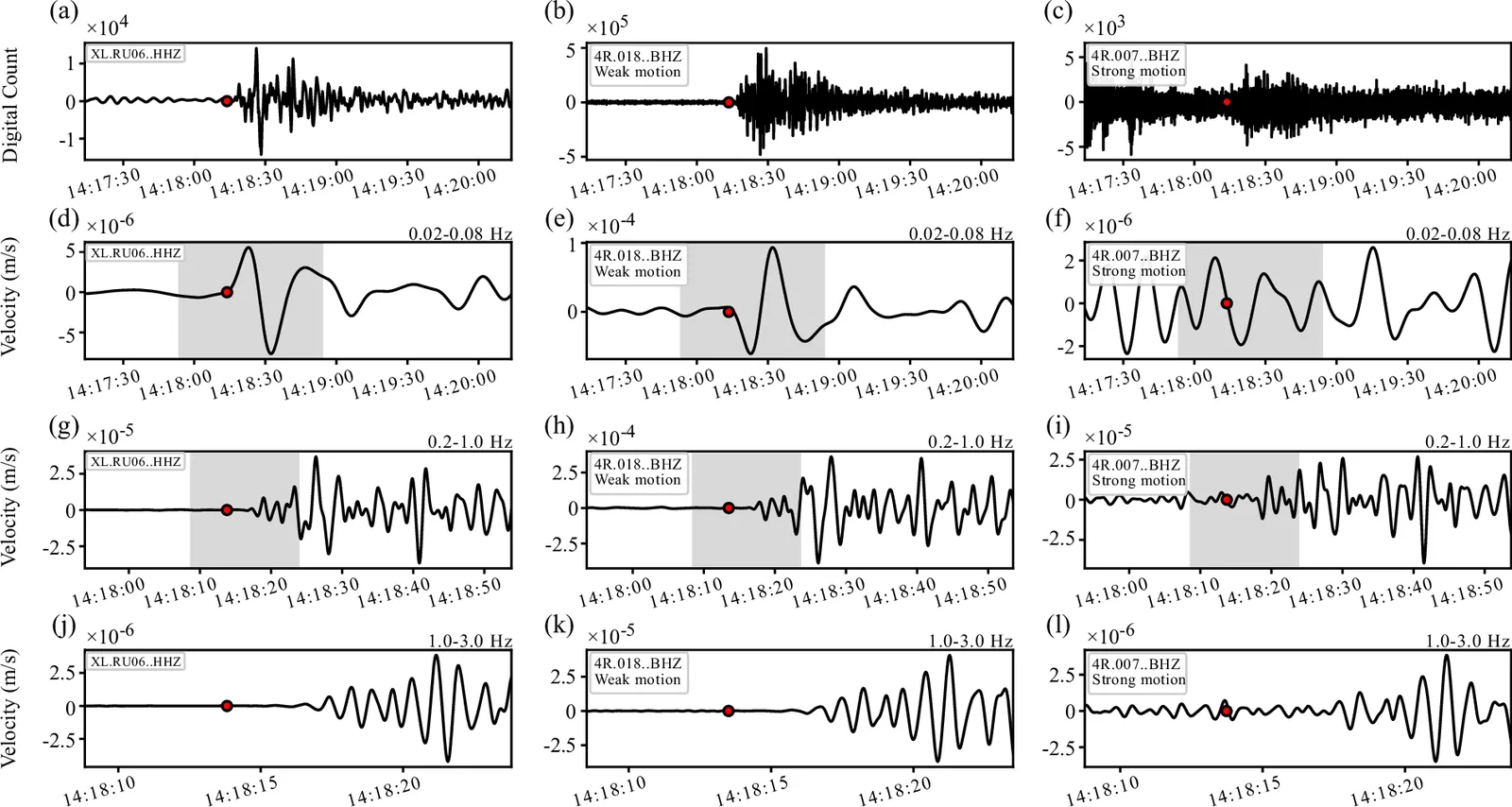
Comparison of raw (top row: panels (a)-(c)) and instrument-corrected (second to fourth rows) vertical-component seismograms recorded at three stations during a teleseismic event. The corrected waveforms are bandpass filtered into three frequency bands (0.02–0.08 Hz: panels (d)-(f); 0.2–1.0 Hz: panels (g)-(i); 1.0–3.0 Hz: panels (j)-(l)) to illustrate differences in sensitivity and signal clarity among the sensor types. Rows 3 and 4 show close-up waveforms of the gray-shaded time windows in rows 2 and 3, respectively. Theoretical *P*-wave arrival times, calculated using the AK135 model^30^, are shown as solid dots. The complete procedure for downloading the waveforms, performing instrument response removal, and applying a bandpass filter is presented in Listing S2 of Supporting Material.

The performance of each sensor is dictated by its design, specifically its instrumental noise level and its frequency response. The comparative behavior of the three instruments reflects the trade-offs built into their analogue front-ends and ADC. The broadband Trillium Compact 20S (i.e., XL.RU06), trimmed for *μ*m/s ground motions, enables long-period teleseismic signals to be recorded well above its self-noise, swinging ±10 kcounts with minimal pre-event noise. The Symroc weak motion sensor (i.e., 4R.018) drives the ADC nearly 1.7 orders of magnitude harder for the earthquake signal, exhibiting the largest peak-to-peak counts (±500 kcounts) on raw seismograms due to its significantly higher instrument sensitivity compared to the other sensors. In contrast, the Symroc strong motion recorder (i.e., 4R.007) is configured to remain linear during large shaking of ±4g, sacrificing its sensitivity to weak motions in order to record very strong motions on-scale and without saturation; therefore, its teleseismic packet is contaminated by gain-independent electronic noise and occupies only a few thousand counts (~±5 kcounts; Fig. 6a–c) .

After deconvolution of instrument responses and band-constrained signal extraction, the contrasting signal-to-noise performance of the three stations follows directly from their analogue transfer functions and intrinsic self-noise. In the 0.02–0.08 Hz band, the Trillium-Compact 20S RU06 sits only 1-16 dB below its passband plateau, so the long period energy is recorded with minimal degradation, while the weak motion sensor 4R.018 is attenuated by ~60–80 dB (Table 2), but after response removal, still exhibits a clear wave train because its low internal noise floor remains well beneath the +60 dB-restored signal. By contrast, the strong motion unit 4R.007 is fixed at approximately −59 dB below its passband throughout this band and its higher baseline noise is boosted by the same factor, leaving phase arrivals submerged (Fig. 6d–f). Between 0.2 and 1 Hz, RU06 is in its flat pass-band and 4R.018 is 15–40 dB below its pass-band, and both yield high-quality *P*-wave onsets, whereas 4R.007, though improved, is still several decibels noisier (about 15–25 dB compared to 4R.018) as it continues to recover from its low front-end gain (Fig. 6g–i). Once the traces are filtered to 1–3 Hz, the strong motion channel (4R.007) climbs off its −59 dB long-period floor and the signal quality improves, but the analogue response is still 16-34 dB below its plateau and its true flat-band conditions are not reached until >~10 Hz, so the 1–3 Hz coda on 4R.007 still remains slightly noisier than on RU06 or 4R.018, which are already within or a few decibels off their passbands in this range (Fig. 6j–l). These observations underscore how each instrument’s amplitude response, combined with self-noise, governs the usable bandwidth for teleseismic studies.

**Table 2:** Velocity-response amplitudes derived from Nanometrics Trillium Compact 20S and Symroc sensors metadata at key frequencies examined in Figs. 6 and 7.

Frequency (Hz)	$| {\boldsymbol{| }}{{\boldsymbol{H}}}_{{\bf{TC20S}}}{\boldsymbol{| }}|$ ($\frac{{\bf{counts}}}{{{\bf{ms}}}^{{\boldsymbol{-}}{\bf{1}}}}$)	$| {\boldsymbol{| }}{{\boldsymbol{H}}}_{{\bf{Weak}}}{\boldsymbol{| }}|$ ($\frac{{\bf{counts}}}{{{\bf{ms}}}^{{\boldsymbol{-}}{\bf{1}}}}$)	$| {\boldsymbol{| }}{{\boldsymbol{H}}}_{{\bf{Strong}}}{\boldsymbol{| }}|$ ($\frac{{\bf{counts}}}{{{\bf{ms}}}^{{\boldsymbol{-}}{\bf{1}}}}$)
0.02	4.79 × 10^7^	1.17 × 10^6^	1.96 × 10^7^
0.08	2.79 × 10^8^	1.85 × 10^7^	1.93 × 10^7^
0.2	2.98 × 10^8^	1.15 × 10^8^	1.81 × 10^7^
1.0	2.99 × 10^8^	2.88 × 10^9^	1.72 × 10^8^
3.0	2.99 × 10^8^	1.60 × 10^10^	1.62 × 10^9^

**Fig. 7:**
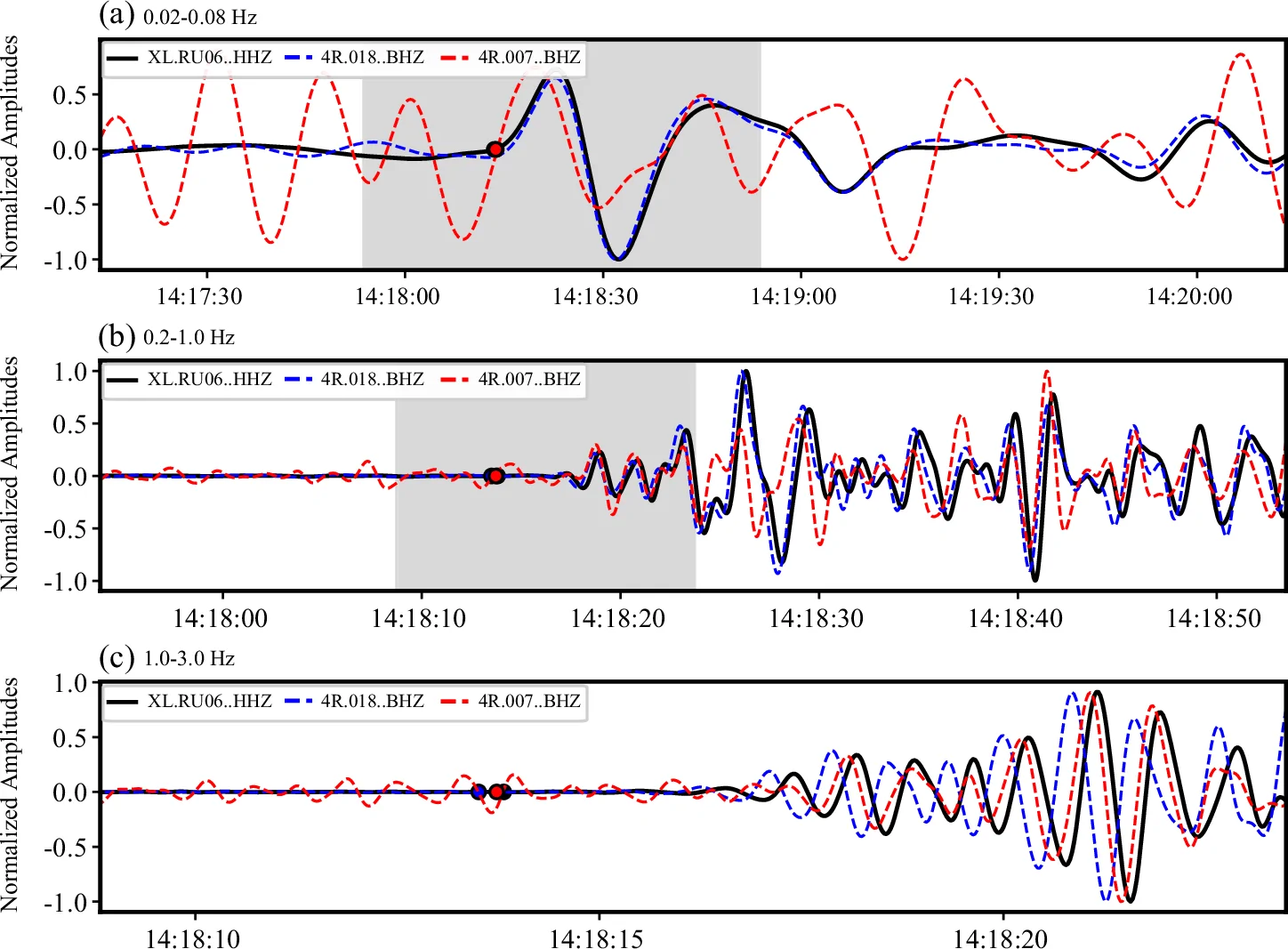
Polarity comparison of normalized vertical-component waveforms across three stations at different frequency bands (0.02–0.08 Hz: panel (a); 0.2–1.0 Hz: panel (b); 1.0–3.0 Hz: panel (c)). The polarities of Symroc waveforms presented here have been reversed relative to their original polarities shown in Fig. 8. This reversal highlights a consistent 180^°^ polarity discrepancy between the Symroc data and Nanometrics Trillium Compact 20S on their vertical channels. Panels (b) and (c) show close-up waveforms of the gray-shaded time windows in panels (a) and (b), respectively. Solid dots show the theoretical *P*-wave arrival times, calculated using the AK135 model^30^, and are color-coded according to station.

Using the teleseismic event as a reference, we compared vertical-component waveforms after instrument-response removal and band-pass filtering (at different frequency bands of 0.02–0.08, 0.2–1, and 1–3 Hz). Across all bands, the Symroc weak and strong motion stations (4R.018 and 4R.007) exhibit a consistent 180^°^ polarity reversal on their vertical channels relative to XL.RU06, indicating a persistent sign-convention or metadata error (e.g., positive-down Z orientation or an inverted overall response) rather than a frequency-dependent phase effect (see Figs. 6 and 7). This systematic polarity inversion should be corrected prior to first-motion picking or waveform-based source-mechanism analysis; otherwise, compressional versus dilatational assignments and moment-tensor estimates will be biased. The clarity of the teleseismic signals enables a straightforward cross-station polarity check among these nearby stations.

**Fig. 8:**
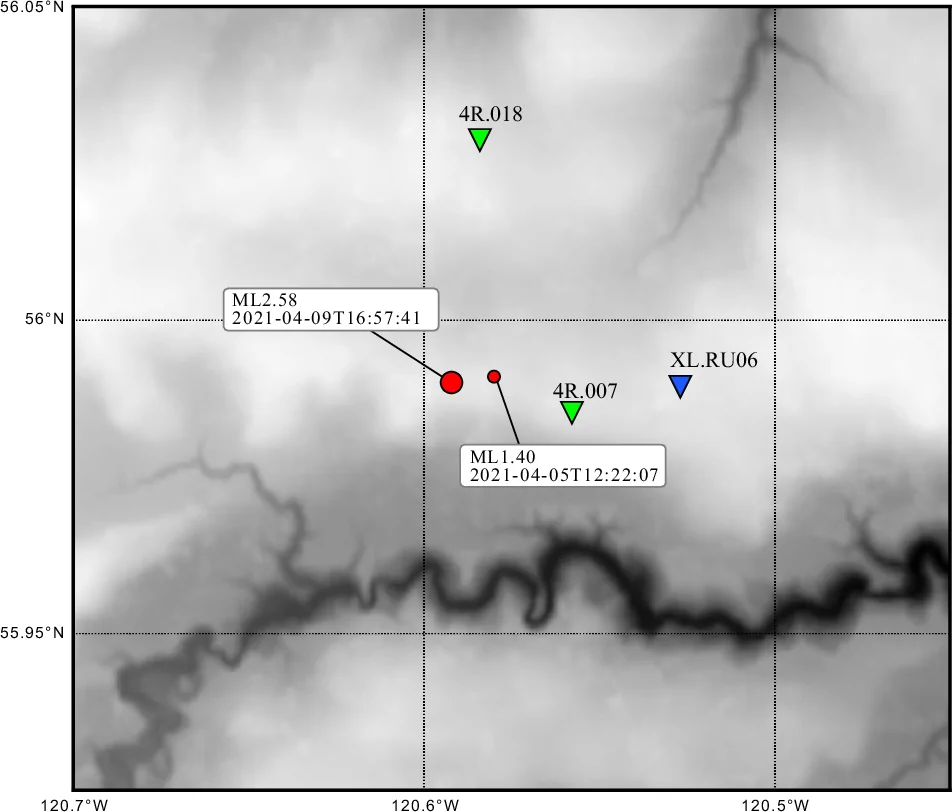
Locations of the two local induced events (M_L_2.58 and M_L_1.40) and the three seismic stations used for high-frequency waveform comparison (see Figs. 9 and 10).

**Fig. 9:**
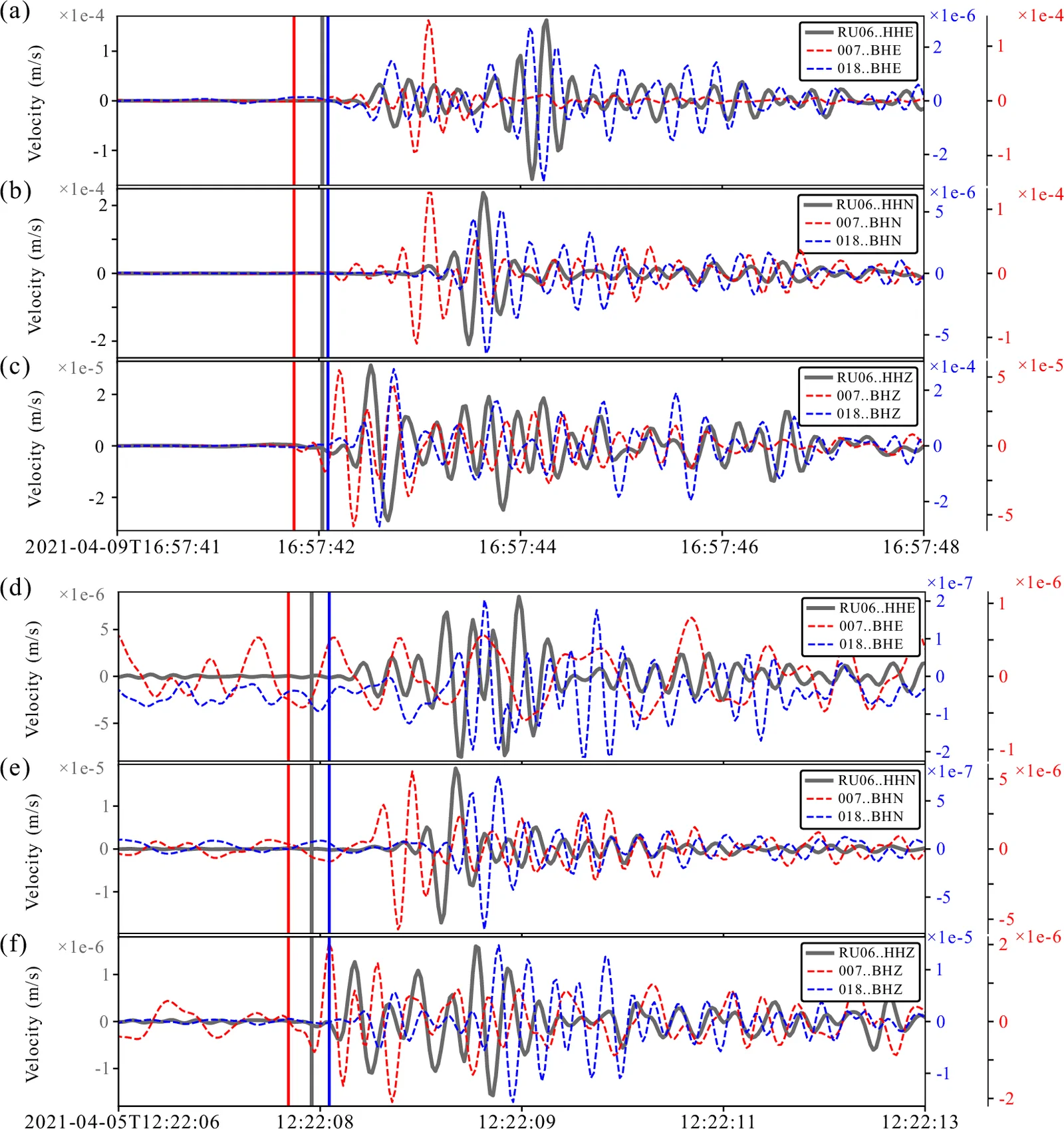
Three-component waveform comparison for two local induced events recorded at the Tower Lake monitoring network. Panels (a)-(c) show the east, north and vertical components, respectively, for an M_L_2.58 event on 2021-04-09T16:57:41 (55.990^°^N, 120.592^°^W) recorded at stations XL.RU06 (gray waveforms), 4R.007 (red dashed waveforms), and 4R.018 (blue dashed waveforms) at respective distances of 3.90, 2.07 and 4.22 km. Panels (d)-(f) show similar waveforms recorded from an M_L_1.40 event on 2021-04-05T12:22:07 (55.991^°^N, 120.580^°^W) at respective distances of 3.10, 1.36 and 4.17 km from XL.RU06, 4R.007 and 4R.018. Waveforms were obtained after removing instrument responses and bandpass-filtered between 1 and 5 Hz. The polarities of Symroc vertical channel waveforms are reversed relative to their original polarities. The *P*-wave arrival onsets (vertical lines) and vertical axis tick labels are color-coded to match their corresponding waveforms. Stations XL.RU06, 4R.007 and 4R.018 are equipped with Nanometrics Trillium Compact 20S, Symroc strong motion, and Symroc weak motion sensors, respectively.

**Fig. 10:**
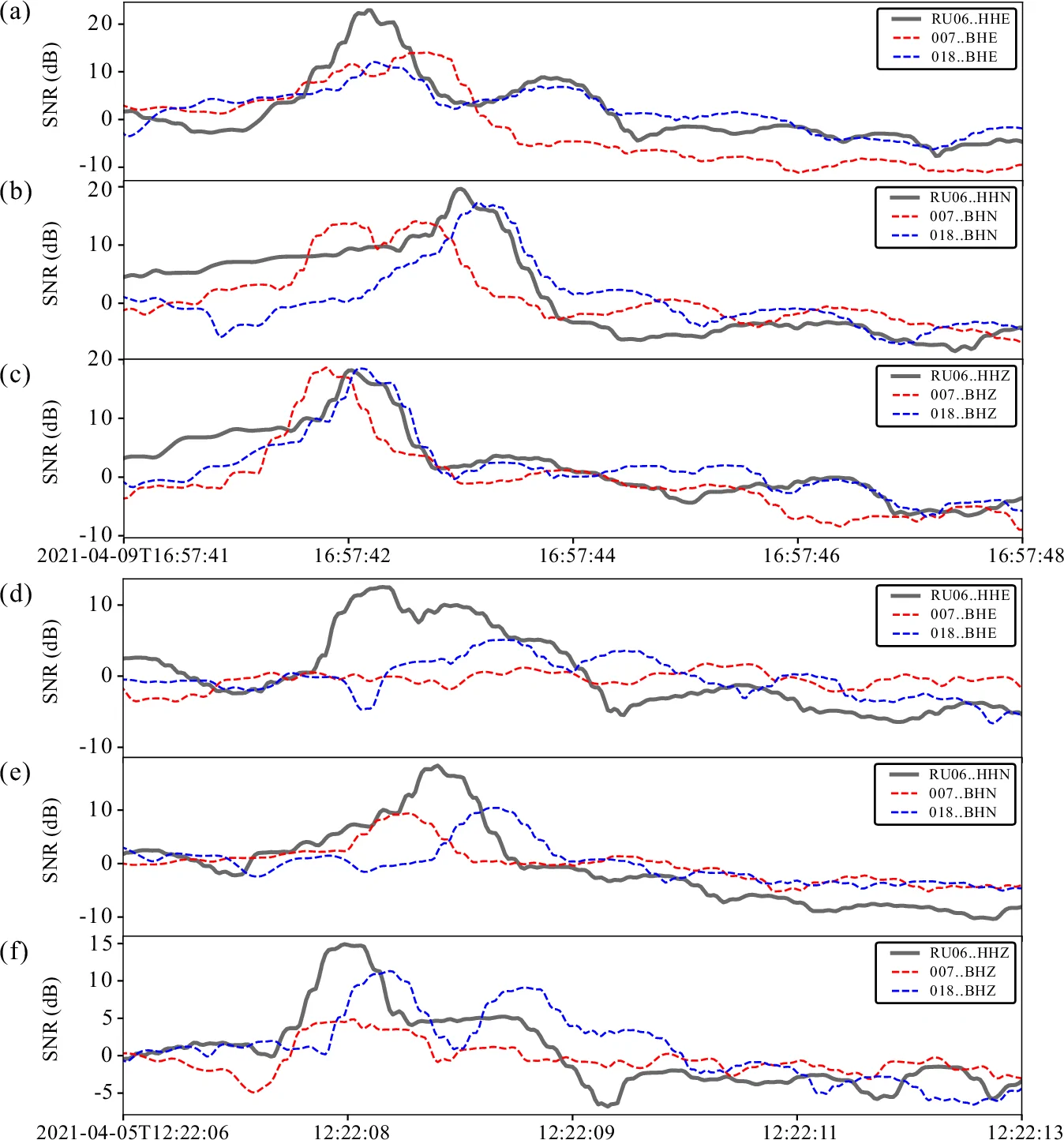
Signal-to-Noise Ratio (SNR) comparison for waveforms recorded from two local events presented in Fig. 8. Displays are analogous to Fig. 8, illustrating SNRs for the M_L_2.58 (**a**–**c**) and M_L_1.40 (**d**–**f**) events. The SNRs are calculated using signal and window lengths of 0.5 and 5.0 s, respectively.

To further investigate the high-frequency sensitivity and detection thresholds of the integrated network, the performance of the broadband, strong motion, and weak motion sensors was evaluated for two local induced events (with M_L_2.58 and M_L_1.40 at distances less than 5 km) within the 1 to 5 Hz frequency range (Fig. 8). For the magnitude 2.58 event, the broadband sensor exhibits a high degree of consistency with the strong and weak motion sensors across all three components, confirming that the array responds uniformly to moderate-magnitude local seismicity (see Fig. 9a–c). This consistency is supported by the corresponding signal-to-noise ratio (SNR) analysis (Fig. 10), where the broadband sensor achieves a peak performance of nearly 20 dB, while the strong and weak motion sensors maintain robust peaks between 10 and 20 dB across all components (see Fig. 10a–c).

As the event magnitude decreases to M_L_1.40, the broadband sensor continues to provide high-fidelity records with SNR peaks greater than 10 dB, despite significant drops in peak velocity amplitudes. In contrast, the strong motion sensor exhibits a significantly reduced SNR, particularly on the east component where the earthquake signal is buried within the noise, though the main phase arrival remains clearly distinguishable on the vertical component. Meanwhile, the noise level of the weak motion data is relatively lower than that of the strong motion data, showing higher SNR values of approximately 5 to 10 dB across all three components (see Figs. 9d–f and 10d–f). Collectively, these observations demonstrate that the monitoring network possesses the necessary sensitivity to identify weak induced events, providing a robust foundation for detailed source characterization.

The TLDA experiment provides a high-resolution dataset for studying the microseismicity response of unconventional reservoirs to fluid injection activity. This high-density monitoring approach offers significant potential for enhancing regulatory measurements and seismic risk mitigation strategies.

## Usage Notes

The TLDA dataset is accessible via standard NSF SAGE and FDSN Web Services, supporting various automated retrieval methods such as the command-line tools like Client URL (cURL). For Linux systems (verified on Ubuntu 22.04.5 LTS), a 10-minute segment of waveform data from station 4R.007 can be retrieved, for instance, using the following request structure:

curl -L -o 4R.007.mseed https://service.iris.edu/fdsnws/dataselect/1/query?net=4R&sta=007&loc=--&cha=BH?&starttime=2021-03-17T00:00:00&endtime=2021-03-17T00:10:00.

## Supplementary information

Supplementary Information

## Data Availability

The TLDA dataset is available at the EarthScope SAGE Data Management Center under the network code 4R (https://ds.iris.edu/mda/4R/?starttime=2021-01-01T00:00:00&endtime=2021-12-31T23:59:59; https://doi.org/10.7914/1yev-vt66; last accessed March 2026).

## Code availability

No custom code was developed for this study. All figures were generated using publicly available software packages, including Python’s ObsPy, Matplotlib, and Basemap, as well as Julia’s GMT package.
