# Supplementary material for: The 2021 Tower Lake Dense Array Experiment for High-Resolution Microseismicity Monitoring During Hydraulic Fracturing
Source: Sci Data. 2026 May 9;13:1596. doi: 10.1038/s41597-026-07358-1 (PMC13759537; doi:10.1038/s41597-026-07358-1)
Supplement: Supplementary file 1 [file 41597_2026_7358_MOESM1_ESM.pdf]

Supporting Material for

The 2021 Tower Lake Dense Array Experiment for High-Resolution Microseismicity Monitoring During Hydraulic Fracturing

Ramin M.H. Dokht, Honn Kao, Ryan Visser, Chet Goerzen, and Stuart Venables

April 20, 2026

This supporting document provides supplementary tables that accompany the primary article on the 2021 Tower Lake Dense Array (TLDA) experiment. The material focuses on technical specifications of the instrumentation used in the study.

The document presents detailed information on the natural and corner frequencies of the sensors, including the linear time-invariant system representations and damping ratios used to characterize the Symroc weak- and strong-motion instruments.

This supporting document includes the following Tables:

S1 Undamped and damped natural frequencies and damping ratios of Symroc sensor responses, derived from the instruments' poles-zers. 2
S2 Station location information for the Tower Lake array (netwrok code 4R). * Weak motion sensors; the remaining stations host strong motion sensors. 3

This supporting document includes the following Listings:

S1 Downloading and plotting instrument response curves for station 4R.018..BHZ using the Python library ObsPy. 4
S2 ObsPy script for seismic data acquisition and processing: downloading the vertical channel waveform (4R.018..BHZ) via FDSN, removing instrument response to velocity (m/s), and applying a 0.02–0.08 Hz bandpass filter. . . 5

Natural and Corner Frequencies

A linear seismometer is modeled as a linear time-invariant system and is fully described in the Laplace domain by its poles (p_m) and zeros (z_k):

$$H(s) \; = \; K \, \frac{\displaystyle\prod_{k=1}^{N_z} (s - z_k)}{\displaystyle\prod_{m=1}^{N_p} (s - p_m)}, \tag{S1}$$

with complex conjugate Laplace pairs $z_k = -\sigma_z \pm j\omega_{dz}$ and $z_m = -\sigma_p \pm j\omega_{dp}$, where σ and ω_d describe the decay rate of the osculation and damped natural angular frequency of the system. K is the overall scalar gain with units output-per-input (e.g., count/(ms^{-1}) for a velocity seismometer), which packages the sensor, preamplifier, and digitizer sensitivities.

The undamped natural angular frequency for any complex Laplace pair, $-\sigma \pm \omega_d$, is defined as:

$$\omega_n \; = \; \sqrt{\sigma^2 + \omega_d^2}, \tag{S2}$$

and its damping ratio is equal to:

$$\xi \; = \; \frac{\sigma}{\omega_n}. \tag{S3}$$

The corner frequency is defined as the frequency at which the response magnitude is -3 dB below the flat part of the gain spectrum; in other words, the response magnitude, $|H(\omega)|$, falls to $\frac{1}{\sqrt{2}}$ of its passband value (or the response power, $|H(\omega)|^2$, reduced by a factor of 2): $10 \log_{10} \frac{1}{2} = 20 \log_{10} \frac{1}{\sqrt{2}} \simeq 3$ dB[1]. For a second-order seismometer that is near-critically damped (i.e., $\xi \simeq 0.7$), the corner frequency, ω_c, corresponds closely to the undamped natural frequency ω_n (Table S1).

Table S1: Undamped and damped natural frequencies and damping ratios of Symroc sensor responses, derived from the instruments' poles-zers.

**Sensor**	**Element**	ω_d (rad)	ω_n (rad)
Weak motion*	Two zeros at s = 0	0	0
	Complex poles at $s = -10.210 \pm 11.937j$	11.937	15.708
Strong motion**	Complex zeros at $s = -1.140 \pm 1.732j$	1.732	2.074
	Complex poles at $s = -43.982 \pm 44.871j$	44.871	62.832

* The damping ratio for the weak motion sensor is $\xi =\sim 0.65$.
** The damping ratio for the weak motion sensor is $\xi =\sim 0.7$.

Table S2: Station location information for the Tower Lake array (netwrok code 4R). * Weak motion sensors; the remaining stations host strong motion sensors.

**Station code**	**Latitude (°)**	**Longitude (°)**	**Elevation (m)**
001	55.9927	-120.571	768.779
002	55.9993	-120.558	756.334
003*	56.0096	-120.544	737.860
004	56.0073	-120.571	749.159
005	56.0143	-120.557	762.122
006	55.9999	-120.587	749.685
007	55.9854	-120.558	777.569
008	55.9924	-120.545	766.294
009	56.000	-120.526	765.973
010	55.9952	-120.519	786.319
011	55.9857	-120.536	776.988
012	55.9779	-120.543	733.077
013	56.0071	-120.597	753.111
014	56.0151	-120.608	779.239
015	56.0146	-120.584	775.427
016	56.0224	-120.596	801.678
017	56.023	-120.571	775.234
018*	56.0289	-120.584	777.992
019	55.972	-120.558	689.155
020	55.9635	-120.565	618.711
021	55.9777	-120.571	709.092
022	55.9713	-120.588	690.492
023	55.9633	-120.594	681.958
024	55.9582	-120.584	640.773
025	55.9854	-120.584	761.562
026	55.98	-120.596	718.736
027	55.975	-120.61	697.63
028	55.9933	-120.595	778.698
029	55.9843	-120.608	784.035
030	55.9776	-120.627	717.916
031	56.000	-120.61	761.803
032	55.9915	-120.621	776.567
033	55.9849	-120.636	767.586
034	56.0036	-120.623	763.173
035*	55.9943	-120.642	811.926
036	56.0025	-120.633	777.96

Listing S1: Downloading and plotting instrument response curves for station 4R.018..BHZ using the Python library ObsPy.

```
from obspy.clients.fdsn import Client

client = Client("IRIS")

# Fetch 4R.018..BHZ inventory
inv = client.get_stations(
    network="4R", station="018", location="", channel="BHZ",
    level="response"
)

# Plot the instrument response
inv.plot_response(min_freq=0.001)
```

Listing S2: ObsPy script for seismic data acquisition and processing: downloading the vertical channel waveform (4R.018..BHZ) via FDSN, removing instrument response to velocity (m/s), and applying a 0.02–0.08 Hz bandpass filter.

```python
from obspy.clients.fdsn import Client
from obspy import UTCDateTime

client = Client("IRIS")

# 1. Download waveforms and station metadata (required for response removal)
network, station, location, channel = "4R", "018", "", "BHZ"
start = UTCDateTime("2021-02-13T14:17:00")
end = UTCDateTime("2021-02-13T14:20:10")

# Download waveforms and the inventory (metadata)
st = client.get_waveforms(network, station, location, channel, start, end)
inv = client.get_stations(
    network=network, station=station, location=location, channel=channel,
    starttime=start, endtime=end, level="response")

# 2. Remove Instrument Response
# output="VEL" (velocity), "DISP" (displacement), or "ACC" (acceleration)
# pre_filt defines a cosine taper to suppress noise outside the instrument's band
pre_filt = [0.001, 0.005, 45, 50]
st.remove_response(inventory=inv, output="VEL", pre_filt=pre_filt)

# 3. Apply Bandpass Filter
# freqmin and freqmax define the passband in Hz
st.filter("bandpass", freqmin=0.02, freqmax=0.08, corners = 4, zerophase = True)

# 4. Plot the processed three-component data
st.plot()
```

References

1. Rodgers, P. W. Frequency limits for seismometers as determined from signal-to-noise ratios. Part 1. The electromagnetic seismometer. *Bulletin of the Seismological Society of America* **82**, 1071–1098 (1992). https://doi.org/10.1785/BSSA0820021071.
